# Treatment with at Homeopathic Complex Medication Modulates Mononuclear Bone Marrow Cell Differentiation

**DOI:** 10.1093/ecam/nep119

**Published:** 2011-02-17

**Authors:** Beatriz Cesar, Ana Paula R. Abud, Carolina C. de Oliveira, Francolino Cardoso, Raffaello Popa Di Bernardi, Fernando S. F. Guimarães, Juarez Gabardo, Dorly de Freitas Buchi

**Affiliations:** ^1^Departamento de Biologia Celular, Setor de Ciências Biológicas, Universidade Federal do Paraná (UFPR), Curitiba, PR, Brazil; ^2^Instituto de Tecnologia do Paraná, Tecpar, Curitiba, Paraná, Brazil

## Abstract

A homeopathic complex medication (HCM), with immunomodulatory properties, is recommended for patients with depressed immune systems. Previous studies demonstrated that the medication induces an increase in leukocyte number. The bone marrow microenvironment is composed of growth factors, stromal cells, an extracellular matrix and progenitor cells that differentiate into mature blood cells. Mice were our biological model used in this research. We now report *in vivo* immunophenotyping of total bone marrow cells and *ex vivo* effects of the medication on mononuclear cell differentiation at different times. Cells were examined by light microscopy and cytokine levels were measured *in vitro*. After *in vivo* treatment with HCM, a pool of cells from the new marrow microenvironment was analyzed by flow cytometry to detect any trend in cell alteration. The results showed decreases, mainly, in CD11b and TER-119 markers compared with controls. Mononuclear cells were used to analyze the effects of *ex vivo* HCM treatment and the number of cells showing ring nuclei, niche cells and activated macrophages increased in culture, even in the absence of macrophage colony-stimulating factor. Cytokines favoring stromal cell survival and differentiation in culture were induced *in vitro*. Thus, we observe that HCM is immunomodulatory, either alone or in association with other products.

## 1. Introduction

Recently, great advances in conventional medicine have included the discovery of powerful new drugs the requested bibliographies such as 2-chloro-2-deoxyadenosine (CdA) for use in patients with various types of cytopenia and opportunistic infections [1], and Decitabine, a cytosine analog used at lower doses for the treatment of myelodysplastic syndrome [2]. In addition, biomarker and drug studies have aided in the development of personalized medicines [3]. Such new medications act to assist rapid patient recovery, but with side effects, which reduce quality-of-life. In this context, Conforti and collaborators in 2007 [4], comment the action of conventional anti-inflammatory drugs and compare with, homeopathic treatment who is used to regulate the pathological excess of inflammation expressing the natural healing dynamics and the efficacy of high dilution of active natural substances. Previous studies demonstrated that a homeopathic complex medication (HCM) activates macrophages (MΦ) both *in vivo* and *in vitro*. It was observed that the *in vitro* production of tumor necrosis factor-*α* (TNF-*α*) by MΦ was significantly reduced when HCM was administered [5]. NADPH oxidase activity increased after HCM ingestion, as did that of inducible nitric oxide synthetase (iNOS), resulting in production of reactive oxygen species (ROS) and nitric oxide (NO), respectively [6]. HCM stimulated the endosomal/lysosomal system and the phagocytic activity of MΦ interacting with *Saccharomyces cerevisiae* and *Trypanosoma cruzi* epimastigotes [7]. The modulatory effects of HCM were also observed both *in vivo* and *in vitro* in experimental infections with *Leishmania amazonensis* and *Paracoccidioides braziliensis;* HCM controlled infection progression and limited pathogen dissemination [8, 9]. Moreover, HCM is neither toxic nor mutagenic [10]. Similarly, improvement in the immune response of mice bearing Sarcoma-180 tumors was seen after HCM treatment. In the cited study, a reduction in sarcoma size was shown and lymphoid cells significantly infiltrated the tumors; granulation tissue and fibrosis surrounded the sarcomas. All animals of the treated group survived, and, in 30% of mice, complete tumor regression was observed. The total number of leukocytes was increased by HCM treatment. Among lymphocyte classes, T-CD4, B and natural killer (NK) cells increased in number [11]. These results suggested that HCM affected hematopoiesis either directly or indirectly. Bone marrow cells were treated with HCM *in vitro* and examined by light, scanning electron, and confocal microscopy. These modalities, and also flow cytometry, indicated that cells of the monocytic lineage (CD11b) and stromal cells (adherent cells) were activated by HCM treatment, which also increased cell clusters over adherent cells, suggesting that cell proliferation and differentiation were taking place [12, 13].

The microenvironment influences growth and differentiation of hematopoietic cells. Adherent cell layers elaborate soluble factors and deposit extracellular matrix which, in turn, influence hematopoietic proliferation and differentiation [14, 15]. The differentiation of monocytic cells into monoblasts, promonocytes, and monocytes (Mo), is stimulated by macrophage colony-stimulating factor (M-CSF) [16]. This factor acts principally to stimulate the proliferation of progenitors committed to MΦ lineages [17–20].

Mo, tissue MΦ, dendritic cells (DCs), microglia and osteoclasts all contribute to maintenance of tissue homeostasis and provide a first line of defense against invading pathogens. These cell types are produced in bone marrow, and undergo differentiation therein before being released to peripheral blood. The recovery of myelopoiesis to normal levels would contribute significantly to increased life-span by preventing delayed severe side effects (e.g., secondary infections) after chemotherapy. Provision to the periphery of more Mo/MΦ, and normalization of bone marrow cellularity, might also permit more intensive patient treatment.

Thus, the aim of this study was to use *in vitro*, *in vivo* and *ex vivo* techniques to examine whether HCM stimulates a preferential response of the Mo/MΦ lineage in the place of origin, the microenvironment surrounding progenitor cells.

## 2. Methods

### 2.1. Animals

Male Swiss mice of the Rockefeller lineage (10–12 weeks of age) were kindly supplied by the Instituto de Tecnologia do Paraná (TECPAR). Animals had free access to food and water. All recommendations of the National Law (No. 6.638; 5 November 1979) for scientific animal management were observed and the Institutional Animal Care Committee of the Universidade Federal do Paraná approved all relevant practices. Experiments were carried out at the Laboratory of Research in Neoplastic and Inflammatory Cells, which has a management program for animal residues.

### 2.2. Animal Treatment

The following groups were established:

 Group 1: control group; mice did not receive any treatment;
 Group 2: mice treated with HCM
 Group 3: mice treated with both HCM and M-CSF;
 Group 4: positive control; mice treated with M-CSF.

Mice were subcutaneously treated at daily intervals for 7 days. Animals receiving HCM were given doses of 7 *μ*L g^−1^ body weight per day. Mice of group 3 received 2.8 *μ*L g^−1^ of HCM and 4.2 *μ*L g^−1^ of M-CSF per day. These amounts were calculated to reflect concentrations used in *in vitro* experiments [9]. After 7 days of treatment animals were euthanized by cervical dislocation and bone marrow cells were obtained as described below. Cells were processed following routine protocols. Experiments were performed at least three times in quadruplicate, independently. A total of 100 animals were used.

### 2.3. Bone Marrow Preparation (In Vitro and In Vivo)

After cervical dislocation femurs were dissected and cleaned. Epiphyses were removed and the marrow was flushed with Dulbecco's Modified Eagle's Medium (DMEM) containing 10% (v/v) fetal bovine serum (FBS) and 1 *μ*g mL^−1^ ciprofloxacin (all from Sigma Pharma, St. Louis, MO). Some cells were used for immunophenotyping and the remaining cells were purified on 1.077 Ficoll-Hypaque (FH) (Sigma). This product is a solution of Ficoll and sodium diatrizoate adjusted to a density of 1.077 g mL^−1^. When blood is overlaid and the solution is centrifuged, mononuclear cells concentrate at the plasma-reagent interface [21]. For FH purification, 10 mL of flushed bone marrow aspirate was layered onto 3 mL of FH solution in a sterile 15 mL centrifuge tube. The tube was capped and centrifuged in a tabletop centrifuge at 1500 g for 40 min at room temperature. A diffuse band of leukocytes (mononuclear cells) formed above the erythrocytes and polymorphonuclear cells, which were together in the pellet. This diffuse cell layer was aseptically removed with a pipette and transferred to a sterile 15 mL centrifuge tube. Cells were washed with phosphate buffered saline (PBS) because Ficoll is toxic to cells. Mononuclear cells were counted in a Neubauer chamber and suspended in DMEM with 10% (v/v) FBS supplemented with 1 *μ*g mL^−1^ ciprofloxacin and 4 mM l-glutamine (Sigma).

### 2.4. The HCM

HCM is a new immunomodulatory therapy and formulation follows Hahnemann's ancient homeopathic techniques. HCM is an aqueous, colorless and odorless solution produced and sold in authorized drugstores in Brazil. Mother tinctures are purchased from agencies authorized by the Brazilian Health Ministry. These agencies assure the quality (endotoxin-free) and physicochemical composition of the product. Starting from the original mother tincture (e.g., a plant ethanolic extract), several dynamizations (succussion, or shaking, and dilution in distilled water) are performed. Decimal dilutions (dH) are prepared. The final commercial product is composed of 11 dH *Aconitum napellus* (Ranunculaceae), 19 dH *Thuya occidentalis* (Cupressaceae), 18 dH *Bryonia alba* (Curcubitaceae), 19 dH *Arsenicum album* (arsenic trioxide), 18 dH *Lachesis muta* (Viperidae), and <1% (v/v) ethanol, in distilled water. We used the commercial product of Narciso da Lozzo Neto, batch number CRF-PR 5604. A homeopathy principle is that substance effects become stronger with dilution and dynamization. Thus, dilution followed by succussion should increase drug potency. The medication was always vigorously succussed immediately before use.

### 2.5. Preparation of L929-Conditioned Medium

M-CSF is highly expressed by the L929 mouse cell line [22] and L929 cell-conditioned medium was used as a source of M-CSF for cells cultured in plastic dishes in DMEM with 10% (v/v) FBS; L-cell conditioned medium was added to 30% (v/v). As M-CSF was not a mother tincture, a high dilution (as used for HCM) was not employed, nor was succussion necessary.

To obtain L929 cell-conditioned medium, L929 cells were seeded in culture bottles (150 cm^2^) to a density of 1 × 10^6^ cells per bottle, and cultivated in DMEM, which is rich in glucose, supplemented with 5% (v/v) FBS. The conditioned medium was collected after 7 days, at which time the cells were confluent. The medium was centrifuged to remove cells and the supernatant was stored at −20°C until use. M-CSF was stable under these conditions for more than 6 months. Once thawed, aliquots were stored at 0°C to avoid the M-CSF degradation that occurs during freeze-thaw cycles.

### 2.6. Liquid Culture (In Vitro)

Cells were adjusted to a concentration of 2.5 × 10^5^/mL, plated in 24-well culture plates, and maintained at 37°C under a 95% air/5% CO_2_ atmosphere for 96 h. All experiments were performed at least three times in quadruplicate; there were four animal groups. Controls received no treatment, because our previous results showed no statistical differences between a control group and an ethanolic aqueous solution group. Cells of group 2 were treated with 20% (v/v) HCM. The M-CSF groups received 30% (v/v) M-CSF medium and, in group 3, cells were treated with both M-CSF medium and 20% (v/v) HCM. Groups 2 and 3 also received 1% (v/v) HCM daily, at precise 24 h intervals.

### 2.7. Cytokine Quantification (In Vitro)

After 96 h of culture adherent cells had increased in number. Supernatant cytokine levels were measured using a mouse Th1/Th2 cytokine CBA kit (BD Pharmingen, USA), according to the manufacturer's instructions. The kit contains antibodies against TNF-*α*, interferon-*γ* (IFN-*γ*), and interleukins 2, 4 and 5 (IL-2, IL-4, IL-5). Cytokine concentrations were obtained by comparison of experimental data with standard curves of the CBA program (Becton Dickinson). Fluorescence was measured using a FACSCalibur flow cytometer (Becton Dickinson), equipped with an argon ion laser (488 nm).

### 2.8. Immunophenotyping

This experiment was preliminary in nature, and was performed to guide further work. After *in vivo* treatment immunophenotyping was performed as soon as cells were collected. Cells (10^6^) were fixed with 1% (v/v) paraformaldehyde, washed, and incubated with 0.5 *μ*g mL^−1^ of the biotinylated antibodies listed below, in PBS for 40 min. Cells were washed with PBS and incubated with 0.5 *μ*g mL^−1^ phycoerythrin (PE)-labeled secondary antibody in PBS for 30 minutes [23]. Fluorescence was analyzed according to standard procedures using a FACSCalibur flow cytometer. Data were analyzed by Cell Quest.

### 2.9. Surface Markers

All antibodies used were from a mouse lineage panel, specific to bone marrow, and were purchased from BD Pharmingen (see Table 1).

### 2.10.Ex Vivo Culture Conditions

Animals were treated as described above and bone marrow cells were collected. Mononuclear cells were purified, concentrated to 2.5 × 10^5^ cells/mL, plated in 24-well culture plates with glass coverslips for adherent cell experiments, and maintained at 37°C under 95% air/5% CO_2_ for 24, 48, and 72 h. Groups 2 and 3 received 1% (w/v) HCM daily, precisely 24 h after the preceding dose.

### 2.11. Morphological Assay

Cells (2.5 × 10^5^) were plated into culture plates with coverslips for morphological analysis [24] and maintained as described above. After 24, 48 and 72 h, cells were rinsed with PBS, fixed in Bouin, stained with Giemsa, dehydrated and mounted with Entellan. Adherent cells were observed by light microscopy using a Nikon Eclipse E200. Structural characteristics of lymphocytes, resident macrophages, activated macrophages, cell niches and cells with ring-shaped nuclei, were sought. On each cover slip, 100 cells were examined. Ten cover slips for each treatment and timepoint were prepared and analyzed. Mean data, in percentages, were transformed as described below.

### 2.12. Statistical Analysis

In cytokines experiments, data were analyzed with Cell Quest software, according to the manufacturer's instructions. ANOVA and the Tukey test (significance: *P* < .05) were used for intergroup comparisons.

Percentage data, obtained from light microscopy analysis, were transformed to $\sqrt{x+0.5}$ values, to give normal distributions. Data were submitted to analysis of variance (ANOVA) with a factorial diagram, randomly delineated, to determine statistical significance. The Tukey test was performed when an interaction effect was significant. The levels of significance were taken to be *P < .05 and **P < .01.

## 3. Results

### 3.1. Cytokine Quantification (In Vitro)

**Figure 1:**
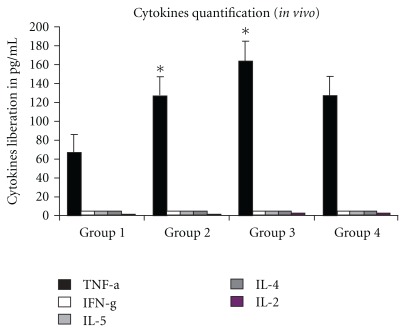
Cytokine quantification *in vitro*. Mice were euthanized and mononuclear cells were obtained by Ficoll separation. Cells were cultured and *in vitro* treated. After 96 h, supernatants were collected and cytokine levels analyzed. These results are presented as mean ± SEM. The significance of differences between mean values was evaluated by two-way analysis of variance (ANOVA) followed by Tukey test. *P < .05 were considered statistically significant. Only the TNF-*α* data are statistically significant at *P < .05. Data are representative of three independent experiments.

After 96 h of culture, we evaluated the capacities of cells to produce TNF-*α*, IFN-*γ*, IL-2, IL-4 and IL-5. TNF-*α* was significantly higher in groups 2 and 3 than in groups 1 and 4 (Figure 1).

### 3.2. Immunophenotyping

**Figure 2:**
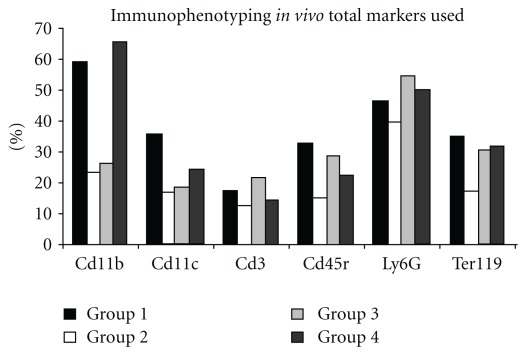
Immunophenotyping. An aliquot was used to analyze the cells just after collecting. Mice were euthanized and bone marrow cells were removed. Aliquots of freshly collected cells were analyzed by flow cytometry. Group 1: control group; Group 2: mice treated with HCM; Group 3: mice treated with HCM and MCSF; Group 4: mice treated with M-CSF. We show data on all markers used in analysis. All bone marrow cells after *in vivo* treatment were subjected to flow cytometry to detect any trend in cell alteration. Decreases in CD11b and TER-119 markers in test groups, compared to controls, may be noted.

After *in vivo* treatment, bone marrow cells were analyzed by flow cytometry. Cell populations were similar to those seen by Civin and Loken in 1987 [25]. The mean percentage of each population is shown in Figure 2. We observed that *in vivo* treatment with HCM decreased the number of CD11b cells (in the Mo lineage). Group 4 (M-CSF treatment) showed more CD11b cells than did group 1 (control); this was expected, as M-CSF is a growth factor specific for this lineage. When HCM was given together with M-CSF (group 3), the data were similar to those of group 2 treated only with HCM.

Dendritic cells (CD11c^+^) were reduced by HCM treatment compared to the control group (group 1). The other groups (3 and 4) showed the same tendency. Granulocytes (Ly6G^+^) and T-lymphocytes (CD3^+^) were apparently downregulated by HCM treatment, but the reduction was very subtle. When HCM was administered together with M-CSF, the reduction was not observed. B-lymphocytes (CD45R^+^) were diminished by HCM treatment in group 2, although groups 3 and 4 also showed slight reductions. Erythrocytes (TER-119^+^) were reduced only in the group treated with HCM alone (group 2).

### 3.3. Morphological Assay

Mononuclear cells obtained from bone marrow of *in vivo* treated animals were observed by light microscopy and characterized by cell morphology. Each cell type was counted, and the data obtained were analyzed statistically. Analysis was performed for each cell type, treatment, and culture time. Adherent cells were characterized as follows.

#### 3.3.1. Lymphocytes

**Figure 3:**
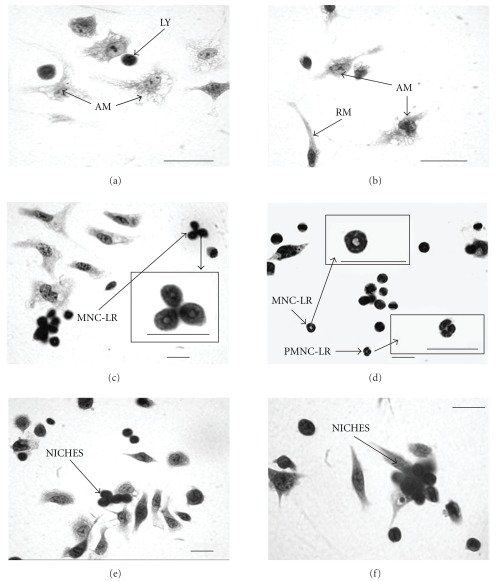
Microphotography of mononuclear bone marrow cells. Animals were treated *in vivo* and bone marrow cells were collected. Mononuclear cells were separated, concentrated to 2.5 × 105 cells/mL, plated on 24-well culture plates with glass coverslips as for the adherent cell experiments, and maintained at 37°C under 95% air/5% CO2 for 24, 48 and 72 h. Cells were stained with Giemsa. Original magnification: 40×. In (c) and (d), we show ring cells and in (e) and (f), we emphasize niches found in groups 2 and 3 after 48 h, respectively. In (a) it is possible to compare lymphocytes (Ly) and activated macrophages (Am) in the positive control (group 4) and in (b) (group 1; the negative control), resident macrophages (Rm) and active macrophages (Am) are seen (Bar = 10 Gm).

**Figure 4:**
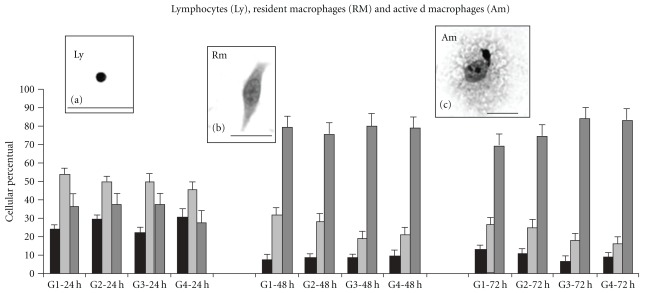
Lymphocytes (Ly), resident macrophages (Rm), and activated macrophages (Ac). Adherent cells were counted at three culture times: 24, 48 and 72 h. The *ex vivo* treatments (G1-group1, G2-group2, G3-group 3 and G4-group 4) showed that lymphocyte and resident macrophage numbers decreased with time of culture. However, activated macrophages had a tendency to increase. These results are presented as mean ± SEM.

Lymphocytes were characterized by their classical morphology, showing rounded small nuclei with condensed chromatin and a thin cytoplasm (Figures 3(a) and 4(a)). After lymphocyte statistical analysis we showed differences only when treatment and culture times were separately compared. Lymphocyte numbers decreased over 48 h and the lower numbers were maintained to 72 h (Figure 4). Group 3 tended to show fewer lymphocytes than did other groups.

#### 3.3.2. Resident MΦ

Resident MΦ were characterized by classical fibroblast-like morphology with a central nucleus, little cytoplasm with few extensions, resulting in an elongated (so not a spread) appearance. Such cells had small, condensed and “kidney"-shaped nuclei (Figures 3(b) and 4(b)). As for lymphocytes, resident MΦ data analysis showed differences only when treatment and culture times were separately compared. We found that group 1 (control) had more resident MΦ (mostly round, with fewer membrane extensions); such cells were rarer in groups 2, 3 and 4. This corroborates previous work showing that HCM activates MΦ, thus decreasing the number of resident MΦ. Although growth factors were absent, we found that after 72 h fewer resident MΦ remained adherent to glass coverslips (Figure 4).

#### 3.3.3. Activated MΦ

The morphological characteristics of activated MΦ are increased membrane ruffling, increased spreading and large and euchromatic nuclei (Figures 3(a), 3(b), and 4(c)). When we examined both treatment and culture time, we observed that the numbers of activated MΦ increased with time in all groups, but there was no significant difference between treatment groups. The addition of HCM, M-CSF, or both, to cultures, increased cell spreading and cell density.

#### 3.3.4. Cells with Ring-Shaped Nuclei

**Figure 5:**
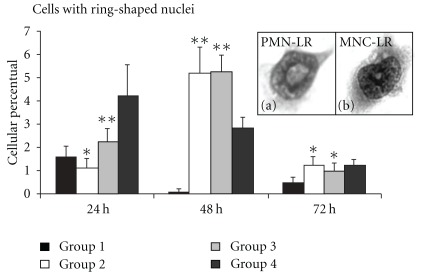
Cells with ring-shaped nuclei (Ring-shaped nuclei cells was removed). These adherent cells were counted. The *ex vivo* treatment showed maintenance of this cell type upon culture and the levels of such cells increased in 48 h of culture. The numbers are perceptual means; *P < .05 and **P < .01. These results are presented as mean ± SEM.

We found cells with constricted ring-shaped nuclei and wide cytoplasmic centers. We observed two such types of cells: polymorphonuclear-like ring cells (PMN-LR) with lobular or constricted ring-shaped nuclei and a cytoplasmic center larger than the width of the ring (Figures 3(d) and 5(a)), and mononuclear-like ring cells (MNC-LR) with ring-shaped nuclei of smooth contour and cytoplasmic centers smaller than the width of the ring (Figures 3(c), 3(d), and 5(b)). With this cell type, ANOVA showed that all interactions were significant and the Tukey test was performed. In groups 2 and 3 these cells appeared at 24 h and increased in number over the next 24 h. After 72 h the numbers of such cells decreased in all groups these dates are showed in Figure 5.

#### 3.3.5. Cell Niches

**Figure 6:**
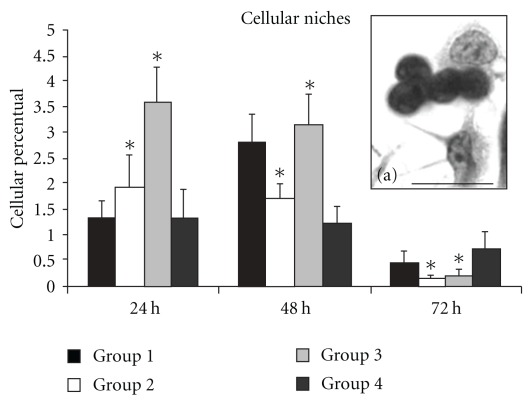
Cellular niches. Cells in niches were counted among adherent cells. We emphasize that this type of cell arrangement was seen immediately upon plating after *in vivo* treatment. The niches were maintained for 48 h, decreasing in number after 72 h. The numbers are perceptual means; *P < .05. These results are presented as mean ± SEM.

Cell niches were associated with adherent layers in small foci over stromal cells (Figures 3(e), 3(f), and 6(a)). After ANOVA analysis we found that all interactions were significant and the Tukey test was performed to compare the means. Groups 2 and 3 showed a higher level of cell niches at 24 h and 48 h, but this decreased with culture time. These dates are showed in Figure 6.

**Figure 7:**
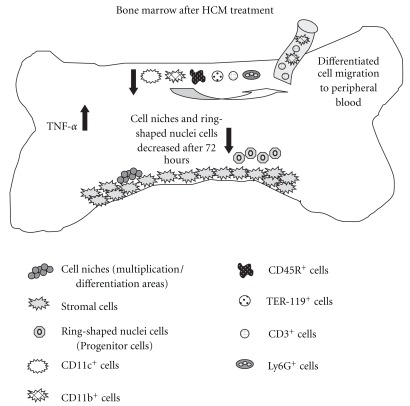
After 96 h of *in vitro* treatment the TNF-*α* concentration has shown a significant increase. After *in vivo* treatment, it was decreased the number of monocyte/macrophages cells (CD11b+), dendritic cells (CD11c+), granulocytes (Ly6G+), B lymphocytes (CD45R+), T lymphocytes (CD3+) and specifically in group 2, erythrocytes (TER-119+). Cell niches and cells with ring-shaped nuclei were present in the first 48 h, and after this diminished.

For better representation of our results, we constructed a schematic representation (Figure 7) to show the effect of the medicament in all aspects analyzed.

## 4. Discussion

Hematopoietic cells proliferate *in vivo* and develop in association with bone marrow stromal cells. Proliferation and differentiation also occur *in vitro*, either in association with stromal cells or in response to soluble growth factors. Our *in vitro* studies have shown that HCM promotes growth and differentiation of myelomonocytic cells in normal mice [12, 13]. Stromal cells produce different types of growth factors leading to the formation of microenvironments that allow cell differentiation into specific lineages.

Homeopathic regulation can be obtained through the similia principle in health body of what constitutes the vital force and its possible dynamic alterations and could be translated in today's terms as both homeodynamic and communication disorders. Bellavite and collaborators [26] mentioned that, when a factor of imbalance occurs a signal is triggered and the system increases its activity producing a greater amount of signal (e.g., IL-1, cytokines, interferons), released from inflammatory exudate, thus bringing the effector system (phagocytes or complement) back to its normal homeodynamic, by eliminating a signal excess and re-establishing condition (healing).

First, several proinflammatory cytokines and chemokines are simultaneously induced by viral and bacterial products in infected or inflamed tissue, and cytokines can co-operate even within a single cell type [27]. Subsequently, host-derived cytokines act synergistically to enhance chemokine induction [28]. Alternatively, some inflammatory chemokines may cooperate with constitutively produced chemokines to influence the distribution of both immature and mature hematopoietic cells, including cell release into the circulation or homing to the bone marrow. Our results showed an increase in TNF-*α* production when HCM was added to cultures. TNF-*α* is an important cytokine with hematopoietic-regulating activities, targeting specific population(s) of bone marrow cells. TNF-*α* has pleiotropic effects on hematopoiesis, depending on the target cells involved, the developmental stage of target cells, or both [29].

Leukocyte production in the bone marrow is controlled by inflammatory cytokines. Acting in concert, TNF-*α* and IL-1 induce the loss of bone marrow lymphocytes by emigration while significantly expanding granulocyte production. Zhang and collaborators, in 1995 [30], established that TNF-*α* is a bifunctional regulator of hematopoiesis. A single dose of the cytokine stimulated the growth of immature murine myeloid progenitors, whereas daily injections induced a slight decrease in such progenitors, accompanied by neutrophilia and lymphopenia. The effects of TNF-*α* on the growth of hematopoietic stem and progenitor cells may change, depending on the phase of the cell cycle. This cytokine can suppress the growth of early hematopoietic progenitor cells *in vivo* and *in vitro*. We found elevated numbers of cells with ring nuclei in groups 2 and 3, suggesting that HCM stimulates the movement of granulocyte precursors, as well as Mo, from bone marrow to the circulation.

Cells with ring nuclei were described as Mo/MΦ lineage precursors by Biermann and colleagues in 1999 [31], not only in rodents, but also in hamsters and humans. Patients with mononucleosis and myeloproliferative disorders also present with these cell types. Different types of cells with ring nuclei were found within 24 h of HCM treatment, including polymorphonuclear-like ring cells (PMN-LR) and mononuclear-like ring cells (MNC-LR). The number of cells with ring nuclei increased significantly after 48 h of treatment, but diminished with time thereafter (Figure 5). Similarly, it is important to note that immunophenotyping showed that the specific granulocytic lineage marker (Ly6G) increased in group 3 (Figure 2). Adhesion, during at least one period of culture, is a feature differentiating Mo/MΦ and PMN cells from other leukocytes. As ring nuclei cells are of myeloid origin and are Mo/MΦ and PMN precursors, we suggest that, after adhesion for a period between 24 and 48 h, differentiated PMN detach to the supernatant. The MΦ precursors remain adherent, increasing the number of activated MΦ in cultures treated for 72 h (Figure 4).

In ours morphological results, we observed cell clusters over the adherent cells (Figure 6), suggesting sites of multiplication and differentiation called cell niches. These niches are known to contain stem cells [32] and/or leukocytes committed with some cellular lineage [33, 34]. The adherent cell clusters are dependent on direct cell-to-cell communication, as well as cytokines and growth factors (colony forming) [35]. In bone marrow erythropoiesis, erythroid cells are organized in small anatomic units termed erythroblastic islets, the islet cells differentiate into cells of the erythroid series, and with MΦ, create these peculiar anatomic units. Soni and co-workers, in 2007 [36], showed that the erythroblast macrophage protein (EMP) is responsible for erythroblast linkage to MΦ on the erythroblastic islets. Each islet is composed of one MΦ surrounded by erythroblasts at different stages of maturation. Mature erythroblasts are considered to move along the cytoplasmic extension of a central MΦ toward the sinusoid [37].

Belon and collaborators, in 2007 [38], mentioned the Arsenicum album administered in homeopathic doses (Arsenicum album-30 and Arsenicum album-200) to revert the alarmingly high incidence of elevated ANA titers observed in random populations of high-risk arsenic contaminated villages in West Bengal, India. Positive modulation was observed along with changes in certain relevant hematological parameters, namely total count of red blood cells and white blood cells, packed cell volume, hemoglobin content, erythrocyte sedimentation rate and blood sugar level, mostly within 2 months of drug administration. A complex homeopathic medicine is produced from *Aconitum napellus, Thuya occidentalis, Bryonia alba, Lachesis muta* and Arsenicum album. It seems to enhance the individual's own immunity to trigger a particular immunologic response against several pathological conditions [5–9, 11].

Even the Immunophenotyping results, were our preliminary experiment, it was demonstrated that HCM treatment decreased expression of the TER-119 marker specific to the erythrocytic lineage (Figure 2). There are two possible explanations for this reduction. Either the maturation process was accelerated by HCM treatment (thus allowing cell migration to the peripheral circulation) or immature cells adhered in niches formed over stromal cells, thus evading detection by flow cytometry. Chen and collaborators [39], genomic approach (microarray analysis) found up-regulated genes are the genes coding for heme hoxygenase HMOX-1 in responses to feverfew extracts. HMOX-1 (heme oxygenase 1, also called HO-1) not only is an essential enzyme in heme catabolism but also plays a critical role in cell migration. HMOX-1 can mediate cell migration by regulating the expression of adhesion molecules. De Oliveira and co-workers [40] have shown that HMOX-1 up-regulated in the microarray analysis.

Cells from the medullar microenvironment and MΦ appear to establish close contact and to form a functional relationship with erythrocytes. This is a key feature in progenitor regulation and differentiation of all lineages, including erythroid precursors [41]. Recent work showed that after HCM treatment such cellular niches could be seen *in vitro* cultures. Scanning electron microscopy demonstrated that MΦ maintained direct contact with the cells, promoting cellular interaction with the extracellular matrix. The contact regions were observed by transmission electron microscopy as adhesion areas between cellular membranes, and the space between any two cells in an adhesion area contained irregularly distributed extracellular particles, forming a septate-like zone with electron-dense points, evidencing the presence of molecules facilitating adhesion between stromal cells [12, 13].

In 1996, Barbé and colleagues [42] showed that the absence of resident MΦ resulted in immature erythrocyte release to the bloodstream. It was also observed that MΦ removal from bone marrow impeded erythroblast adhesion, corroborating the idea that MΦ are important in erythroblast adhesion and differentiation. The identification of niches in the present paper shows that HCM may immunomodulate medullar erythropoiesis caused by MΦ and stromal cells. The arrangement of cells in niches (islets) over MΦ may be important for iron turnover, hemoglobin synthesis, erythropoietin production, and phagocytosis of expelled erythroblastic nuclei [33].

## 5. Conclusion

Our results show that *in vitro*, *in vivo* and *ex vivo* treatment with HCM stimulated cytokines important in cell differentiation and survival of specific monocytic lineage cells and their precursors. We hope these data will find clinical application as a safe and feasible pharmacological treatment of isolated mononuclear cells, to enhance therapeutic efficacy.

## Figures and Tables

**Table 1:** 

Antibody	Principal cell types marked
CD11b (Mac-1)	Monocytes/macrophages
Ly-6G	Granulocytes
CD45R	B-lymphocytes
CD11c	Dendritic cells
CD3	T-lymphocytes
TER-119	Erythrocytes
